# Importance of Tryptophan in Transforming an Amphipathic Peptide into a *Pseudomonas aeruginosa*-Targeted Antimicrobial Peptide

**DOI:** 10.1371/journal.pone.0114605

**Published:** 2014-12-10

**Authors:** Xin Zhu, Zhi Ma, Jiajun Wang, Shuli Chou, Anshan Shan

**Affiliations:** Institute of Animal Nutrition, Northeast Agricultural University, Harbin, China; Indian Institute of Science, India

## Abstract

Here, we found that simple substitution of amino acids in the middle position of the hydrophobic face of an amphipathic peptide RI16 with tryptophan (T9W) considerably transformed into an antimicrobial peptide specifically targeting *Pseudomonas aeruginosa*. Minimal inhibitory concentration (MIC) results demonstrated that T9W had a strong and specifically antimicrobial activity against *P. aeruginosa*, including antibiotic-resistant strains, but was not active against *Escherichia coli*, *Salmonella typhimurium*, *Staphylococcus aureus* and *Staphyfococcus epidermidis*. Fluorescent spectroscopic assays indicated that T9W interacted with the membrane of *P. aeruginosa*, depolarizing the outer and the inner membrane of bacterial cells. Salt susceptibility assay showed that T9W still maintained its strong anti-pseudomonas activity in the presence of salts at physiological concentrations, and in hemolytic and MTT assays T9W also showed no toxicity against human blood cells and macrophages. In vivo assay demonstrated that T9W also displayed no toxicity to Chinese Kun Ming (KM) mice. Furthermore, the strong antibiofilm activity was also observed with the peptide T9W, which decreased the percentage of biomass formation in a dose-dependent manner. Overall, these findings indicated that design of single-pathogen antimicrobial agents can be achieved by simple amino acid mutation in naturally occurring peptide sequences and this study suggested a model of optimization/design of anti-pseudomonas drugs in which the tryptophan residue was a conserved element.

## Introduction

The emergence of multidrug resistant microbes has stimulated research on the development of alternative antibiotics. Antimicrobial peptides (AMPs), which are secreted by the innate immune system of various multicellular organisms as defense against pathogenic invasion, are currently under consideration as alternatives to conventional antibiotics [1]–[4]. To date, approximately 1/3 to 1/2 of the AMPs that have been identified assume a predominant α-helical structure in membrane-mimetic environments [5]. One of the key structural parameters pertaining to helically folded AMPs is the presentation of the amphipathic face upon folding. The amphipathicity of the AMPs is often analyzed by projecting the amino acid residues on a “helical-wheel diagram”. The diagram shows that the charged residues are located on the polar face, while the hydrophobic residues are located on the hydrophobic face of an AMP [6]. Thus, it appears that the polar face of an AMP contributes to the initial electrostatic attraction to a negatively charged cell membrane, while the hydrophobic face contributes to the antimicrobial and cytotoxic activities of AMPs.

It is not surprising, based on the amphipathic structure, considering that the target of AMPs is indeed the cell membrane, and AMPs can usually discriminate prokaryotic organism from eukaryotic organism, given the membrane composition difference between them. This membrane-interactive antimicrobial activity can often be enhanced by changing the hydrophobicity and/or optimizing the integrity of the hydrophobic face of an AMP by substituting hydrophobic amino acid residues [6], [7]. Therefore, these broad-spectrum AMPs have been considered to be as potential antimicrobials. However, these small-molecule antimicrobials have always been killing benign and pathogenic organisms indiscriminately, which often leads to severe antibiotic-associated infections due to the vacated niche available for opportunistic pathogens colonization [8]. The problems resulting from broad-spectrum antibiotic use, combined with the emergence of drug-resistant strains, highlight the urgent and fundamental need for developing new “targeted” AMPs to dissociate the pathogens from the indigenous microflora [9].

Considering the amphipathic structure and mechanism of action of AMPs described above, we believe that new “targeted” AMPs which can specifically distinguish and kill pathogens from otherwise nonpathogenic microbes can be potentially engineered by simple substitution of the amino acid residues in the strategic positions of the hydrophobic or hydrophilic face of the amphipathic AMPs, and the results from recent studies have given us some inspiring clues and implications. Chen et al. observed that the anti-pseudomonas activity was enhanced by simply replacing the central hydrophobic amino acid residue on the hydrophobic face of the amphipathic peptide [10]. Lee et al. also observed that placement of a positive charge on the hydrophobic face was important for anti-pseudomonas activity and modes of action of the AMPs [11]. Recently, we have described an amphipathic peptide, RI16, truncated from the N-terminal of PMAP-36. This peptide formed a helical structure with a wide polar face and a narrow hydrophobic face upon membrane binding. A modification to RI16 based on the α-helical protein framework and folding principles and sequence changes by substituting paired charged amino acids linked by H-H bonding on the polar face of the helix with tryptophan proved to be broad-spectrum against Gram-negative and Gram-positive bacteria and more cell selective towards bacteria than mammalian cells [12]. However, the amphipathic nature of RI16 is disrupted by a polar residue (Thr-9) that falls in the middle position of the hydrophobic face of the amphipathic helix, as demonstrated by the fact that the middle position of the α-helical AMPs is strategic and important in designing and/or optimizing AMPs [10], [13]. To address the role of this strategic and important position and the effects of disrupting or non-disrupting the hydrophobic face of the helical peptide, we have prepared Lys- (T9K) and Ile- (T9I) substituted mutants in which the strict amphipathic helical amino acid distribution is disrupted and recovered, respectively. In addition, aromatic amino acids Trp and Phe-substituted mutants (T9W and T9F) were also considered, as these amino acids are frequently found in natural AMPs and contribute to rendering the peptide to further insertion of the residues into the lipid membrane [14], [15]. In doing so, we employ a method combination of spectroscopic circular dichroism (CD), fluorescent spectroscopy, confocal microscopy, and combine this with determination of the biological activity of peptides, aimed at elucidating effects of the hydrophobic face integrity of helix on function and modes of action of amphipathic peptides. Furthermore, salt susceptibility, in vitro and in vivo cytotoxicity of the lead peptide are also investigated. Here, we find that the integrity of the hydrophobic face of the amphipathic helical peptides has little effects on antimicrobial activity and cytotoxicity of the peptides. Surprisingly, the results highlight a significant role for tryptophan of the hydrophobic face in transforming an amphipathic peptide into a *P. aeruginosa*-targeted AMP, and also the importance in membrane binding and perturbation.

## Materials and Methods

### Peptide synthesis

The peptides designed in this study as well as the fluorescent labeled peptides were obtained from GL Biochem Corporation (Shanghai, China) and were synthesized by solid-phase methods using N-(9-fluorenyl) methoxycarbonyl (Fmoc) chemistry. The peptides were amidated at the C-terminus. The purity (>95%) of the peptides was analysed by reversed-phase high-performance liquid chromatography (RP-HPLC), and the peptides were further subjected to mass spectrometry (MS) to confirm the molecular weight.

### Circular dichroism (CD) spectra

CD spectra of the peptides were measured at 25°C using a J-720 spectropolarimeter (Jasco, Japan). The peptides with a final concentration of 150 µM were dissolved in 10 mM PBS (pH 7.4), 50% trifluoroethanol (TFE), or 30 mM sodium dodecyl sulfonate (SDS). The solutions were loaded into a rectangular quartz cell with a 0.1-cm path length, and the spectra were recorded between 190 and 250 nm at 0.1 nm increments. The average mean residue ellipticities were potted against the wavelength.

### Antimicrobial assays

Minimal inhibitory concentration (MIC) assays were performed as described previously [12]. Briefly, after incubation overnight in Mueller-Hinton (MH) broth at 37°C, the bacteria were grown to exponential growth phase and diluted to approximately 1×10^5^ CFU/ml. The peptides were dissolved in 0.2% bovine serum albumin (BSA) containing 0.01% (v/v) acetic acid, and they were then added to each well of a 96-well plates at a final concentration ranging from 0.25 to 128 µM. Each well contained a total volume of 100 µL (50 µL of inoculums and 50 µL of peptide-containing solution). The MICs were determined as the lowest concentration of peptide that prevented visible turbidity by visual inspection after incubation at 37°C for 16–18 h. Independent experiments were carried out at least three times. Uninoculated MH broth was used as a negative control and cultures without added peptides served as the positive control. Minimal bactericidal concentration (MBC) assay was performed following MIC assay. At the end of MIC assay 10 µL aliquots of the medium were taken from peptide-containing wells with no visible bacterial growth. These are plated on MH agar, incubated for 18 h to allow colony growth. The MBC was defined as the lowest peptide concentration resulting in no bacterial growth in the media. Each assay was performed at least three times.

### Outer membrane permeability assay

The ability of the peptides to depolarize the bacterial outer membrane was evaluated using the fluorescent dye N-phenyl-1-naphthylamine (NPN) as previously described [16]. In brief, *P. aeruginosa* ATCC27853 cells were suspended to give a final OD_600_ of 0.05 in 5 mM HEPES buffer (pH 7.4) containing 5 mM glucose. NPN was added into a bacteria suspension at a fixed concentration of 10 µM. The stabilized background fluorescence was recorded at an excitation wavelength of 350 nm and an emission wavelength of 420 nm. The peptide was added to the quartz cuvette to give the final concentration, and the fluorescence reading was recorded immediately upon the addition of peptide which leads to the maximal increase in NPN uptake.

### Inner membrane permeability assay

The inner membrane depolarization of the peptides was measured using the cyanine 3, 3′-dipropylthiadicarbocyanine iodide (diSC_3_-5) as previously described [17]. Briefly, the bacteria were suspended in HEPES buffer (containing 20 mM glucose, pH 7.4) containing 0.2 mM EDTA to give a final OD_600_ of 0.05. The cell suspension was incubated with a final concentration of 0.4 µM diSC_3_-5 for 60 min in dark. Then, KCl was added to a final concentration of 0.1 M to equilibrate the K^+^ levels. The peptides were added to achieve different final concentrations. Changes in fluorescence were recorded using an F-4500 fluorescence spectrophotometer (HITACHI, Japan) with an excitation wavelength of 622 nm and an emission wavelength of 670 nm.

### Confocal laser scanning microscopy

To analyse the cellular distribution of the peptides, *P. aeruginosa* 27853 were incubated in the presence of FITC-labled peptide and observed on a confocal laser scanning microscopy. *P. aeruginosa* cells (OD_600_ = 0.2) were incubated with peptides at 1×MIC at 37°C. After incubation for 1 h, the cell pellets were collected by centrifugation at 5,000 g for 5 min and washed three times with PBS buffer. A smear was made, and images were captured using a Leica TCS SP2 confocal laser scanning microscope with a 488 nm band pass filter for FITC excitation.

### Susceptibility assays

For salt susceptibility, different salts were employed at their physiologic concentrations: 150 mM NaCl, 4.5 mM KCl, 6 µM NH_4_Cl, 8 µM ZnCl_2_, 1 mM MgCl_2_, and 4 µM FeCl_3_. [18] The MIC determination was conducted as described above.

### Toxicity evaluation

The in vitro cytotoxicity of the peptide against erythrocytes and macrophage cells was determined. Human red blood cells (hRBCs) were obtained from healthy donors (Xin Zhu and Zhi Ma) that voluntarily went to the analysis for a blood routine check-up, after informed verbal consent. This verbal consent was considered to be sufficient because the samples were handled anonymously and were used only to isolate erythrocytes. This verbal consent was approved by the Northeast Agricultural University Hospital Research Ethics Committee. The procedure of use of hRBCs for in vitro experiments was approved by the institutional review board of the Northeast Agricultural University Hospital. The erythrocytes were harvested via centrifugation at 1000 g for 5 min and washed three times with PBS (pH 7.4), and resuspended in PBS to attain a dilution of approximately 1% (v/v) relative to the erythrocyte volume initially collected. Then, 50 µl of the diluted hRBCs solution was incubated with 50 µl of serially diluted peptides dissolved in PBS for 1 h at 37°C. The intact erythrocytes were pelleted by centrifugation at 1000 g for 5 min at 4°C and the supernatant was transferred to a new 96-well microtiter plate. The release of hemoglobin was monitored by measurement of the absorbance at 492 nm. As negative and positive controls, hRBCs in PBS and 0.1% Triton X-100 were employed, respectively.

The MTT assay was performed according to a previously described method [16]. Briefly, 1.0×10^4^ J774.1 macrophage cells/well in Dulbecco modified Eagle medium (DMEM) supplemented with L-glutamine (Gibco) and 10% fetal calf serum (Eurobio) were placed into 96-well plates and then incubated under a fully humidified atmosphere of 95% air and 5% CO_2_ at 37°C overnight. The next day, the peptides were added to the cell cultures at final concentrations of 1 to 128 µM. After incubation for 24 h, the cell cultures were incubated with MTT (50 µl, 0.5 mg/ml) for 4 h at 37°C. The cell cultures were centrifuged at 1,000×g for 5 min, and the supernatants were discarded. Subsequently, 150 µl of dimethyl sulfoxide was added to dissolve the formazan crystals formed, and the OD was measured using a microplate reader (Tecan GENios F129004; Tecan, Austria) at 492 nm.

The in vivo toxicity of the peptide was also evaluated. Female KM mice (weighing 20 to 25 g) were injected intraperitoneally with 0.1 ml saline and 3, 6, 30, and 60 mg peptide per kg of body weight. The animals (10/group) were directly inspected for adverse effects, such as weight loss, piloerection, motility and mortality for 7 days thereafter. All mice were allowed free water and a maintenance diet in a 12-hour light/dark cycle. All cages contained wood shavings and bedding. The experiment was carried out in strict accordance with the recommendations of the Guide for the Care and Use of Laboratory Animals of Northeast Agricultural University, and the study protocol was approved by Northeast Agricultural University Institutional Animal Care and Use Committee. The mice were monitored for a week and no mice died during the experiment.

### Biofilm biomass assay and imaging

A static abiotic solid surface assay was used to analyze biofilm formation as described before [19], with minor modification. Briefly, overnight cultures of *P. aeruginosa* ATCC27853 were diluted 1∶100 in fresh medium, added (500 µL) to wells of 12-well culture plates, and grown for 24 h at 37°C. Planktonic cells and loosely attached cells were removed, and wells were then refilled with 500 µL of fresh broth in the absence or presence of different concentrations of peptides. After incubation the biofilms were stained with 0.1% (w/v) crystal violet. Ethanol (95%, v/v) was added to each well, and absorbance was measured at 600 nm. The experiments were done at least three times in duplicate, and the data was expressed as mean ± standard deviations.

For microscopic observation of the biofilm, sterilized coverslips were placed at the bottom of a 12-well plate. After incubation, cells were fixed with 2.5% glutaraldehyde overnight. The samples were then dehydrated through a graded series of ethanol (50%, 70%, 90%, and 100%), followed by critical–point drying, gold sputtering, and examination using an FE-SEM (Hitachi, Japan).

## Results

### Peptide design and characterization

**Figure 1:**
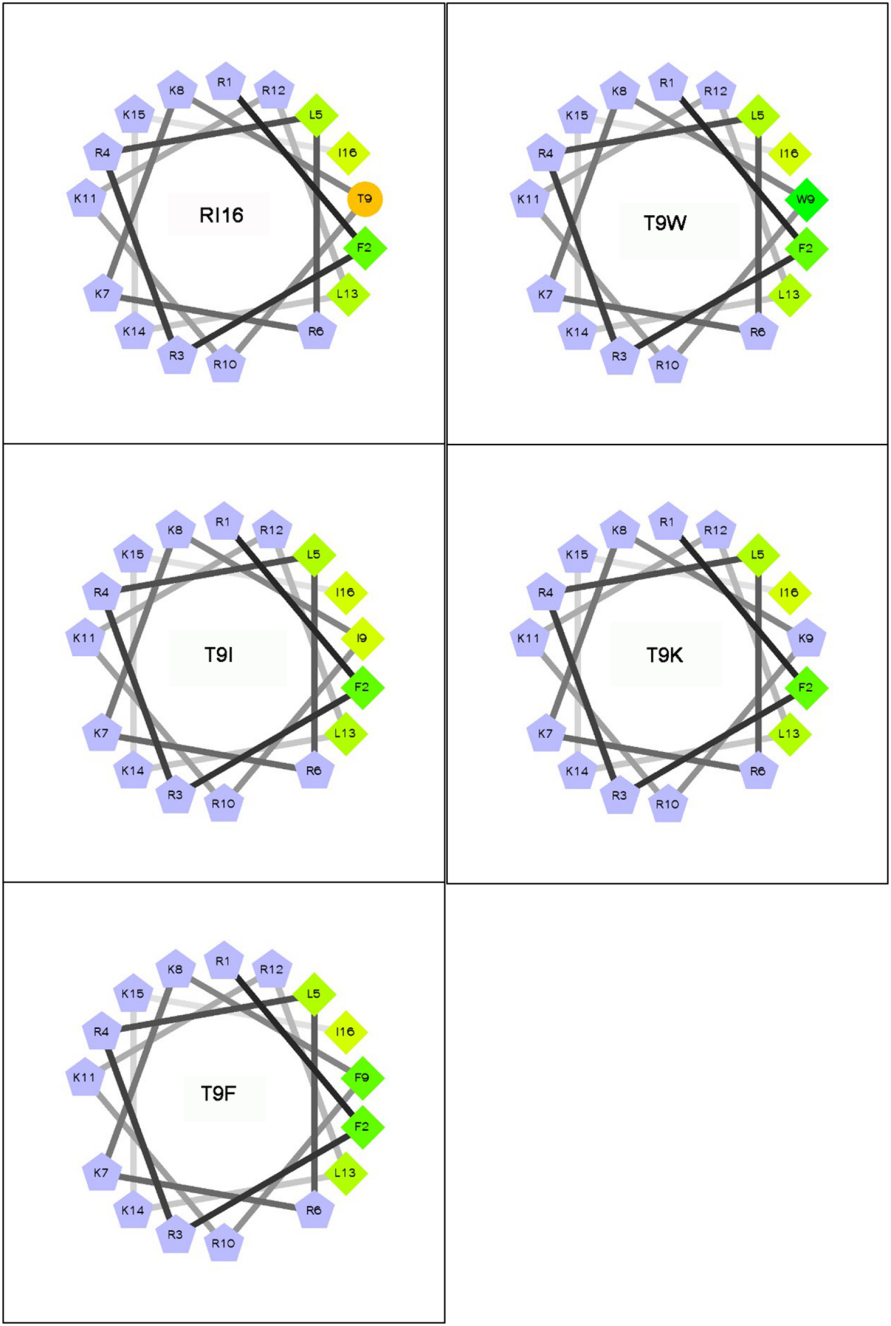
Helical wheel projections of the peptides. By default the output presents the hydrophilic residues as circles, hydrophobic residues as diamonds, potentially negatively charged as triangles and potentially positively charges as pentagons. Hydrophobicity is color coded as well: the most hydrophobic reside is green, and the amount of green decreases proportionally to the hydrophobicity, with zero hydrpphobicity coded as yellow. The positively charged residues are grey and the hydrophilic residues are coded orange. The wheel projection was performed online using the Helical Wheel Projections: http://rzlab.ucr.edu/scripts/wheel/wheel.cgi.

The molecular weight of the peptides was verified by MS. The theoretically calculated and measured molecular weights of each peptide were summarized in Table 1. Each peptide was observed to have a measured molecular weight value that is in very close agreement with its theoretical value, suggesting that the peptides were successfully synthesized. The RI16 and its mutant peptides showed to be highly charged (net charges≥12) and hydrophilic (H<0) (Table 1). The wheel-diagram showed that all the hydrophilic amino acid residues of these peptides are located on one side, whereas the hydrophobic amino acid residues are on the other side of the helix (Fig. 1). The hydrophobic moment values of RI16 (0.615) was higher than that of T9K (0.537), but lower than that of the other mutant peptides T9I (0.711), T9F (0.710) and T9W (0.739), indicating the amphipathic order: T9K<RI16<T9F<T9I<T9W (Table 1).

**Table 1:** Key physicochemical parameters of parental and mutant peptides.

Peptides	Sequence	Theoretical MW	Measured MW^a^	Net charge	H^b^	µH^b^
RI16	RFRRLRKK***T***RKRLKKI-NH_2_	2182.7	2183.7	12	−0.235	0.615
T9W	RFRRLRKK***W***RKRLKKI-NH_2_	2267.8	2267.9	12	−0.111	0.739
T9I	RFRRLRKK***I***RKRLKKI-NH_2_	2194.8	2194.9	12	−0.139	0.711
T9K	RFRRLRKK***K***RKRLKKI-NH_2_	2209.8	2209.9	13	−0.313	0.537
T9F	RFRRLRKK***F***RKRLKKI-NH_2_	2228.8	2228.9	12	−0.139	0.710

a Measured by MS.

b H, hydrophobicity; µH, hydrophobic moment. Calculated from http://heliquest.ipmc.cnrs.fr/cgi-bin/ComputPararmsV2.py.

### Secondary structure

**Figure 2:**
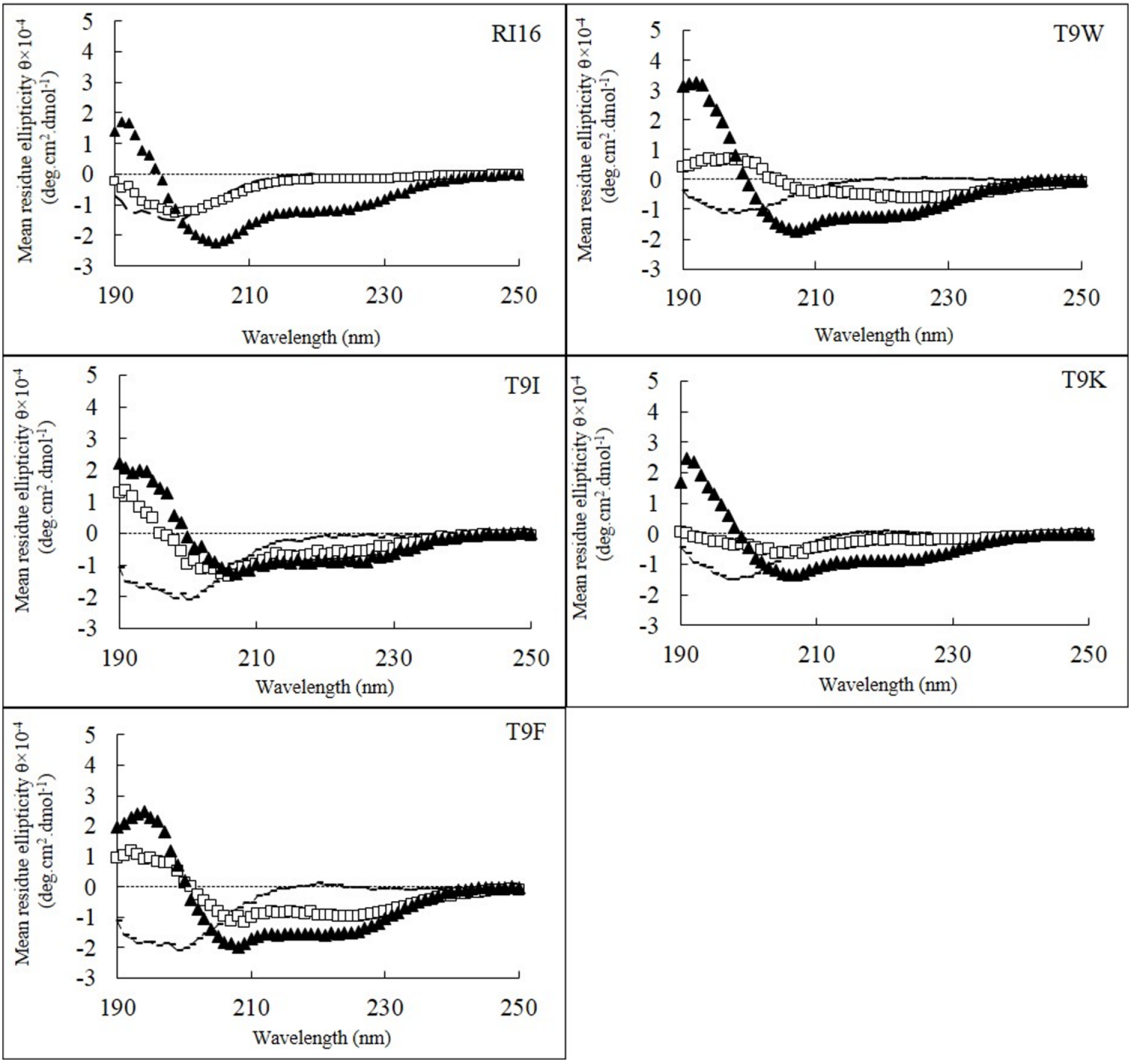
The CD spectra of the peptides. The peptides were dissolved in 10 mM PBS (pH 7.4) (dashed lines), 50% TFE (triangles), or 30 mM SDS (squares). The mean residue ellipticity was plotted against wavelength. The values from three scans were averaged per sample, and the peptide concentrations were fixed at 150 µM.

The spectra of the RI16 and mutant peptides were recorded using CD. The CD spectra of all peptides are characteristic of a random structure in PBS buffer and a helical structure in TFE (Fig. 2). In SDS, the parent peptide RI16 and the mutant peptide T9K still showed the characteristic of a random structure, while the other mutant peptides (T9W, T9F and T9I) exhibited a helical structure.

From Table 2, all peptides exhibited negligible α-helical contents (<6%) in PBS than that in TFE (>58%). However, in SDS, differences in helical contents of peptides were observed. As shown in Table 2, the parent peptide RI16 and the mutant peptide T9K exhibited lower α-helical content (<14%) than other mutant peptides (>38%), and T9F showed the most helical structure (62%). These results indicated that the non-disrupted hydrophobic face along the non-polar face of the peptide stabilizes the helical structure, and the extent of the change in structure depends on the nature and position of the amino acid that is substituted and the environment that the peptide is in.

**Table 2:** CD data of the peptides.

Peptides^a^	PBS		SDS		TFE	
	[θ]_222_ b	% helix^c^	[θ]_222_	% helix	[θ]_222_	% helix
RI16	−113	1	−1670	11	−11784	78
T9W	−212	1	−6010	40	−12369	82
T9I	−728	5	−5849	39	−9073	60
T9K	−337	2	−1960	13	−8986	59
T9F	−533	4	−9443	62	−15123	100

a The peptides (150 µM) were dissolved in 10 mM PBS (pH 7.4), 50% TFE, or 30 mM SDS.

b The mean residue molar ellipticities [θ]_222_ (degree•cm^2^•dmol^−1^) at wavelength 222 nm were measured at 25°C.

c The helical content (%) of a peptide relative to the molar ellipticity value of peptide T9F in 50% TFE.

### Antimicrobial activity

The antimicrobial activity of the RI16 and mutant peptides against Gram-negative and Gram-positive bacteria were determined. Most importantly and notably, T9W showed specific and strong activity, whereas RI16 and other mutant peptides were inactive, against *P. aeruginosa* (Table 3). Meanwhile, both RI16 and mutant peptides showed no significant activity against both Gram-negative bacteria *E. coli* and *S. tryhimurium* and Gram-positive bacteria *S. aureus* and *S. faecium*. For evaluating the anti-pseudomonas activity of the lead peptide T9W further, antibiotic-resistant variants of *P. aeruginosa* were developed by step-wise culture with selected antibiotics at sub-MICs for at least 30 generations. As shown in Table 3, T9W maintained the strong activity against ciprofloxacin-, gentamincin- or ceftazidime-resistant *P. aeruginosa*, while these antibiotics lost their activity against these resistants. Determination of the MBC values indicated that T9W was 1- to 2-fold higher than their MIC values against *P. aeruginosa* (Table 3). Combined with MIC values, it would thus appear that the peptide T9W exerts a bactericidal rather than a bacteriostatic activity against *P. aeruginosa*. Based on the CD results, such observations confirmed the weak correlations between the secondary structure and the activity and the important role of the tryptophan in that the antimicrobial activity of the amphipathic peptide RI16 can be transformed into species-specific.

**Table 3:** Antimicrobial activity and cytotoxic activity of parental and mutant peptides.

Items	MIC^a^ (µM)						MBC^a^ (µM)
	RI16	T9W	T9I	T9K	T9F	Amikacin	T9W
*E. coli* 25922	128	64	128	128	64	0.125	>128
*S. typhimurium* 7731	128	128	256	256	128	0.5	>128
*S. aureus* 29213	128	128	256	128	128	1	>128
*S. epidermidis* 12228	32	64	32	128	32	0.125	>128
*P. auruginosa* 27853	256	2	256	128	256	1	2
*P. auruginosa* 10419	256	4	256	256	256	2	4
*P. auruginosa* 21625	256	4	256	256	256	16	4
*P. auruginosa* 21630	256	4	256	256	256	1	4
*P. auruginosa* LC	32	1	64	256	64	0.25	2
*P. auruginosa* LCCI^b^	256	2	256	256	256	8	4
*P. auruginosa* LCGE^c^	256	4	256	256	256	>1280	8
*P. auruginosa* LCCE^d^	256	4	256	256	256	8	8
Hemocytes (HC_50_ e)	>256	>256	>256	>256	>256	>256	>256
Macrophage (LD_50_ f)	>256	>256	>256	>256	>256	>256	>256

a Minimal inhibitory concentration (MIC) was determined as the lowest concentration of the peptides that inhibited bacteria growth, and minimal bactericidal concentration (MBC) was the concentration resulting in no bacterial growth.

b A ciprofloxacin-resistant strain, which MIC for ciprofloxacin was above 512 µM.

c A gentamincin-resistant strain, which MIC for gentamincin was above 512 µM.

d A ceftazidime-resistant strain, which MIC for ceftazidime was above 512 µM.

e HC_50_ value was taken as the concentration of peptide producing 50% hemolysis.

f LD_50_ value was taken as the concentration of peptide producing 50% cell death.

### Membrane permeability

**Figure 3:**
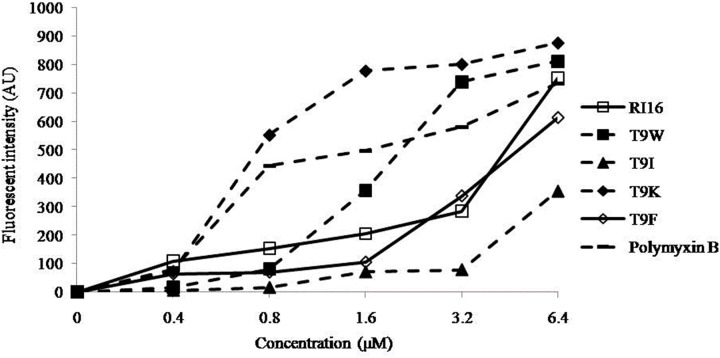
The outer membrane permeability of the peptides. The uptake of NPN of *P. aeruginosa* 27853 in the presence of different concentrations of the peptides determined using the fluorescent dye (NPN) assay. The NPN uptake was monitored at an excitation wavelength of 350 nm and an emission wavelength of 420 nm.

**Figure 4:**
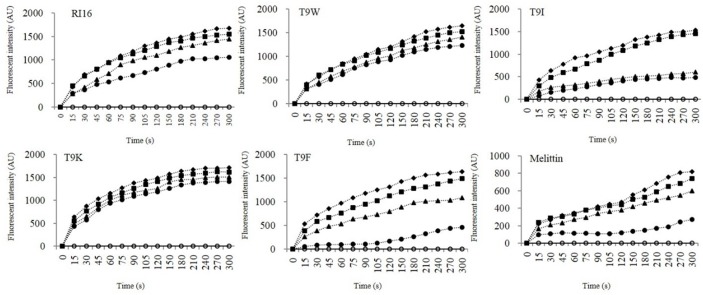
The cytoplasmic membrane potential variation of *P. aeruginosa* 27853 treated by peptides at concentrations of 0 (open circles), 0.8 (solid circles), 1.6 (triangles), 3.2 (squares), and 6.4 (rhombus) µM, as assessed by release of the membrane potential-sensitive dye diSC_3_-5. The fluorescent intensity was monitored at an excitation wavelength of 622 nm and an emission wavelength of 670 nm as a function of time.

NPN uptake and diSC_3_-5 release assays were employed to evaluate the ability of the peptides to permeabilize the outer and inner membranes, respectively. NPN is a hydrophobic fluorescent probe that fluoresces weakly in an aqueous environment and strongly when it enters the interior of a membrane [20]. All peptides were able to cause depolarization of the outer membrane of *P. aeruginosa* at concentrations well below their MICs, and the most cationic peptide T9K significantly induced a more progressive increase of fluorescent intensity than other mutant peptides (Fig. 3). This finding was consistent with that for many cationic peptides that the initial interaction of the peptide with bacterial membrane was predominantly governed by electrostatic forces and cationicity is apparently beneficial for this outer membrane-peptide interaction [5], [21]. The membrane potential-dependent probe diSC_3_-5 will be released into the medium upon dissipation of the membrane potential by permeabilization of the inner membrane [22]. T9W was able to cause rapid depolarization of the inner membrane of *P. aeruginosa* at low concentrations than RI16 (Fig. 4). Moreover, in contrast to T9F, release of diSC_3_-5 was not dependent on the concentration of T9W. These observations suggested the importance of the tryptophan residue on membrane binding and disturbing.

### Confocal laser scanning microscopy (CLSM)

**Figure 5:**
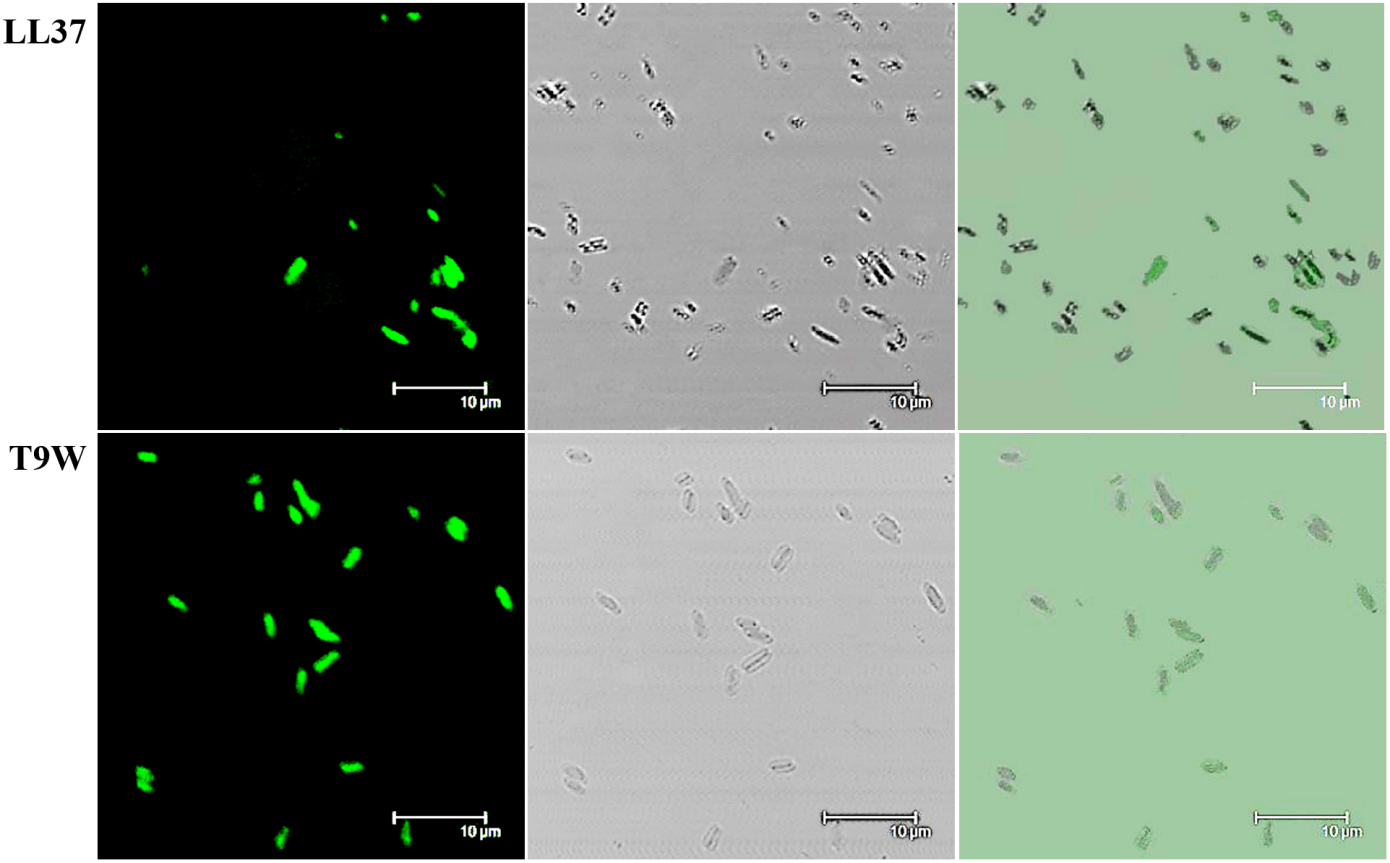
Confocal fluorescence microscopic images of *P. aeruginosa* 27853 cells treated with FITC-labeled peptides at 37°C for 60 min. Panels on the left, middle and right represent laser-scanning, transmitted light-scanning, and merged images of cells treated with FITC-labeled peptides, respectively.

The location of fluorescently labeled peptide in the treated *P. aeruginosa* cells was monitored using confocal laser scanning microscopy. As shown in Fig. 5, T9W accumulated to a higher degree at the cell surface than LL37, the only host defense peptide encoded by the human cathelicidin gene, which eliminates bacteria by targeting membranes [23], [24]. This further suggested that T9W was able to damage the *P. aeruginosa* cells by membrane-peptide interactions.

### Salt susceptibility

In light of the evidence supporting the antimicrobial activity and membrane disruption ability of T9W against *P. aeruginosa* in vitro, it was important to know whether this peptide could retain its anti-pseudomonas efficacy in the presence of salts and whether it would be toxic to mammalian cells. One of multiple obstacles to the development of AMPs for clinical applications is a significantly reduced antibacterial potency in the presence of physiological salts [25]. In this study, physiological concentrations of salts exhibited no or only a partial effect on the anti-pseudomonas activity of T9W (Table 4).

**Table 4:** MIC values of T9W against *P. aeruginosa* 27853 in the presence of physiological salts^a^.

Peptides	Control	Na^+^	K^+^	NH_4_^+^	Mg^2+^	Zn^2+^	Fe^3+^
T9W	2	2	4	2	8	2	2

a The values represent the MICs of T9W in the absence or presence of salts, and the concentrations of the salts were as follows: 150 mM NaCl, 4.5 mM KCl, 6 µM NH_4_Cl, 8 µM ZnCl_2_, 1 mM MgCl_2_, and 4 µM FeCl_3_.

### Cytotoxicity

To be useful in therapeutic application, AMPs need to be selective for prokaryotic cells than mammalian cells. In vitro cytotoxic assays demonstrated that the peptide T9W had no effects on the viability of hemocytes and macrophage cells (Table 3). Likewise, the in vivo toxicity of T9W was also determined, and each group of ten mice showed no signs of toxicity, such as weight loss, piloerection, motility, and all survived for at least 7 days after an intraperitoneal injection of T9W at 3, 6, 30 or 60 mg per kg of body weight, respectively (data not shown). These results suggest that the lead peptide T9W can serve as a promising template for developing therapeutic agents against *P. aeruginosa* infections.

### Antibiofilm activities

**Figure 6:**
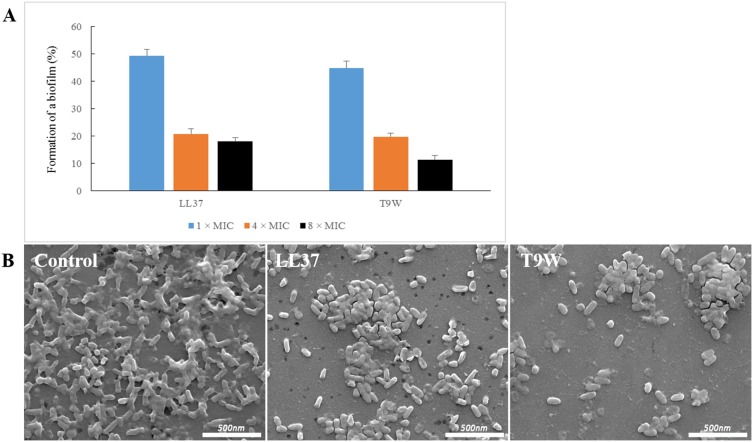
Antibiofilm activities of the peptides. A) Biomass of *P. aeruginosa* 27853 biofilms were reduced after 24 h treatment with various concentrations of LL37 and T9W relative to the untreated control. B) FE-SEM images of *P. aeruginosa* 27853 biofilms treated with the peptides at their 4×MICs.

*P. aeruginosa* is an opportunistic pathogen responsible for numerous infections. Persistent *P. aeruginosa* infections is linked to the formation of a biofilm [26], a complex community of microorganisms attached to a surface and enclosed in a self-produced extracellular matrix [27], [28]. We next determined the antibiofilm ability of T9W on pre-formed *P. aeruginosa* biofilms, which are intrinsically more challenging than the prevention of biofilm formation. As shown in Fig. 6A, T 9W was found to induce a significant reduce in the amount of biofilm biomass in a dose-dependent manner, comparable to LL37, which was previously demonstrated to inhibit biofilm formation [29]. The changes in the amount of biomass after treatment with T9W and LL37 were further investigated under FE-SEM. As shown in Fig. 6B, the cell densities in the biofilms were significantly reduced at 4×MIC concentrations of peptides. These results, taken together, provide direct evidence that T9W is able to efficiently mediate the dispersion of biofilm matrices.

## Discussion

Previous studies demonstrated that the N-terminal portion of PMAP-36 displayed a well-defined amphipathic residue arrangement, with an unusually wide and cationic polar sector and a narrow hydrophobic sector interrupted by the presence of a moderately polar threonine [30], [31]. One of the key structural parameters pertaining to the activity of the helically folded AMP is the presentation of the facial amphipathicity upon folding [6]. Thus the threonine at position 9, which dispersed or disrupted the hydrophobic face of the α-helical peptide, may be a key to the biological activity of PMAP-36. Here, we substituted threonine with lysine (T9K) and isoleucine (T9I) to test the effect of the disrupted and non-disrupted hydrophobic face on the structure-function relationship of the peptides, and phenylalanine (T9F) and tryptophan (T9W) substitutions were used to test the importance of aromatic residues.

It has been demonstrated that the parent peptide RI16 is inherently amphipathic and presents an amphipathic structure [12]. Our results indicated that all the mutant peptides (T9W, T9K, T9I and T9F) were unstructured in PBS, but presented α-helical structures in the presence of 50% TFE, a mimic of hydrophobicity and the α-helix-inducing ability of the membrane, suggesting that these mutant peptides have a comparable potential to form amphipathic and helical structures compared to the parent peptide RI16. In order to further investigate the proclivity of the conformational structures of the peptides in anionic environment, SDS micelles were used as a model lipid system to simulate peptide-lipid interactions, because these detergent like molecules had been reported to form micellar rings around the transmembrane helical region of a membrane protein, similar to the structure of the natural bilayer membrane [32]. We showed here that the peptides with non-disrupted hydrophobic face (T9I, T9W and T9F) exhibited the higher helical structures than that with disrupted hydrophobic face (RI16 and T9K), suggesting the importance of hydrophobicity and/or integrity of hydrophobic face in maintaining the α-helical structure of an amphipathic peptide.

Generally, an increase in hydrophobicity and amphipathicity increased the membrane-activity of the peptides against bacterial and mammalian cells [5]. The results obtained from this study showed that all synthetic peptides were not toxic to hRBCs and macrophages, suggesting that the ability to form a helix or the helicity of peptides in a neutrally hydrophobic environment (TFE) is correlated with cytotoxicity. On the other hand, although the peptides with disrupted hydrophobic face (RI16 and T9K) exhibited lower helical contents than that with non-disrupted hydrophobic face (T9I, T9W and T9F) in an anionic environment (SDS), they all had comparable antimicrobial activity against Gram-negative bacteria *E. coli* and *S. typhimurium* and Gram-positive bacteria *S. aureus* and *S. epidermidis*, indicating that peptide amphipathicity is not always positively correlated with its antimicrobial activity. Previously, Chen *et al.* have demonstrated that the replacing the residue in the middle position on the hydrophobic face of α-helical AMPs with a single charged amino acid has excellent potential for the rational design of AMPs with enhanced activity and specificity [10].

Electrostatic interactions and amphipathic structures are important for the activity of AMPs, but obviously this cannot explain the differences in the relative efficiency of the mutant peptides against *P. aeruginosa*. All designed peptides presented a helical amphipathic structure and displayed similar helical content in the membrane-mimetic environment, and they had a similar net charge (+12 for T9W, T9F and T9I, and +13 for T9K), which appears to be sufficient for the initial electrostatic interactions between the AMPs and the negatively charged bacterial membrane, thus binding and depolarizing the outer membrane of *P. aeruginosa*, as observed in Fig. 3 and Fig. 5. Even with the non-disrupted hydrophobic face or perfect amphipathicity, T9I did not have a strong ability of depolarizing the inner membrane of *P. aeruginosa* than RI16 and T9K with disrupted hydrophobic face (Fig. 4). Even with the same charge and comparable amphipathicity (Table 1), T9W had better anti-pseudomonas activity than T9F. This result implies the particular importance of the tryptophan residue, in agreement with previous studies showing that the tryptophan residue not only partitions more favorably into the membrane interface, but it is also more hydrophobic and has a higher affinity for bulky hydrophobic phases than other aromatic residues [33]. However, the exact action site of membrane by T9W will be necessary to determine, and the process involving peptide-membrane interactions or other intracellular processes cannot also be ruled out.

In light of the evidence supporting the antimicrobial activity and membrane disruption ability of T9W against *P. aeruginosa* in vitro, it was important to know whether this peptide could retain its anti-pseudomonas efficacy in the presence of salts and whether it would be toxic to mammalian cells. One of multiple obstacles to the development of AMPs for clinical applications is a significantly reduced antibacterial potency in the presence of physiological salts [18]. In this study, physiological concentrations of Mg^2+^ slightly inhibited the antimicrobial activity of T9W against *P. aeruginosa*, while the presence of other salts exhibited no or only a partial effect on the anti-pseudomonas activity of T9W. This antagonistic effect of Mg^2+^ on the antimicrobial activity of T9W was mainly attributed to the competition with AMPs for a specific binding site on the surface of the bacterial cells, similarly to what has been observed for other peptides [18], [34], [35].

In vitro cytotoxicity assays demonstrated that the peptide T9W had no effects on the viability of hemocytes and macrophage cells. Likewise, the in vivo toxicity of T9W was also determined, and a group of ten mice all survived for at least 7 days after an intraperitoneal injection of T9W at up to 60 mg per kg of body weight. However, the in vivo efficacy of T9W against *P. aeruginosa* needs to be evaluated further.

Furthermore, as *P. aeruginosa* infection was frequently associated with clinically significant biofilm, the antibiofilm activity of T9W was also determined. Biofilm represents a complex bacterial lifestyle adaptation that presumably allows microbial cells to survive in hostile environments [36]. It has been estimated that biofilm cells are up to 1,000 times more resistant to most antimicrobial agents than planktonic cells [37]. In addition, microbial biofilms are also resistant to host immune mechanisms, thus leading to bacterial infection and hindering treatment [37], [38]. Our results indicated that T9W could effectively disperse *P. aeruginosa* biofilm, showing promise as a potential therapeutic agent against biofilm infections caused by *P. aeruginosa*.

An unexpected finding in this study is the observation that substitution of Thr of RI16 with Trp significantly enhances antimicrobial activity against *P. aeruginosa*, and this enhanced activity is species-specific, but not broad-spectrum. Usually, Trp residue is found in high proportion in many AMPs and always plays important roles for activity. [14] Thus, many studies have mainly involved the modification/optimization of naturally occurring AMPs or the de novo design of simple model AMPs utilizing Trp residue to obtain molecules with optimal biological activity [15], [33], [39]–[44]. Studies of AMPs have suggested that the indole side chain of Trp is ideally suited for interacting with the polar-nonpolar interface, with the highly hydrophobic aromatic ring preferentially buried in hydrophobic region of the lipid bilayer, contributing to the broad-spectrum antimicrobial activity [18], [45]. However, it is not clear that whether AMPs, like broad-spectrum conventional antibiotics, may result not only in antibiotic-induced disease but also have more problematic and lasting consequences of drug-resistance. Throughout evolution, bacteria have devised several mechanisms to protect themselves from deleterious damage of AMPs, as reviewed by Tomaz Koprivnjak et al. (2011) [46]. So, there is a growing consensus that species-specific compounds are the future of antimicrobial agent discovery and development [9], [47]. Indeed, if an agent is active only against a particular pathogen, it is unlikely to have a probability of being toxic to humans, as a target harboured by a particular bacterial species is unlikely to be present in mammals, and also can minimize the likelihood of perturbing the gut symbionts balance and inducing resistance [48]. Although the biological significance of T9W is limited in this study, the finding is very important as it provides information for AMP optimization or design where a more species-specific activity is desired and available.

In summary, we have shown the key role of tryptophan in transforming an amphipathic peptide into a *P. aeruginosa*-targeted antimicrobial peptide by simply substituting a threonine residue on the middle position of the hydrophobic face with a tryptophan residue, and also the importance in membrane binding and perturbation. The lead peptide T9W was also demonstrated to be active against antibiotic-resistant *P. aeruginosa*, resistant to salt susceptibility, not toxic against mammalian cells, and efficient to mediate biofilm dispersion, suggesting the potential for development of anti-*P. aeruginosa* drugs and therapeutic applications.
